# Epitope mapping of a neutralizing antibody against rabbit hemorrhagic disease virus GI.2

**DOI:** 10.1186/s13567-025-01505-z

**Published:** 2025-04-02

**Authors:** Ana Podadera, Mila Leuthold, José Manuel Martín-Alonso, Rosa Casais, Angel Luis Álvarez, M. J. Lobo-Castañón, Francisco Parra, Kevin Paul Dalton

**Affiliations:** 1 Instituto Universitario de Biotecnología de Asturias, Departamento de Bioquímica y Biología Molecular, Edificio Santiago Gascón, Universidad de Oviedo, Campus El Cristo, 33006 Oviedo, Spain; 2 Present Address: Chemistry and Biochemistry Department, University of Windsor, 401 Sunset Avenue, Windsor, ON N9B 3P4 Canada; 3 Medicinal Chemistry, Institute of Pharmacy and Molecular Biotechnology, University of Heidelberg, 69120 Heidelberg, Germany; 4 SERIDA, Servicio Regional de Investigación y Desarrollo Agroalimentario, Centro de Biotecnología Animal, 33394 Gijón, Asturias Spain; 5 Departamento de Química Física y Analítica, Universidad de Oviedo, Av. Julián Clavería 8, 33006 Oviedo, Spain; 6 Instituto de Investigación Sanitaria del Principado de Asturias, Avenida de Roma, 33011 Oviedo, Spain

**Keywords:** mAb-2D9/RHDV GI.2, virus neutralization, RHDV GI.2, P-domain, VLP

## Abstract

In 2010, rabbit hemorrhagic disease virus (RHDV) GI.2 emerged, and unlike RHDV GI.1, it caused mortality in young rabbits, while existing vaccines were not fully protective. The GI.2-specific monoclonal antibody (mAb) 2D9 has been used as a tool to discriminate between these viruses in diagnostic tests. In this study, we mapped the binding epitope for 2D9 on the GI.2 The VP60 capsid protein demonstrated the neutralizing capacity of this mAb, which was able to prevent GI.2 infections in an experimental challenge. Our results suggest that external loops (1, 4 and 5) in the P2 subdomain of VP60 contribute to the discontinuous neutralizing epitope recognized by mAb 2D9. Moreover, analysis of naturally occurring RHDV GI.2 isolates revealed key residues involved in mAb 2D9 binding that are under selective pressure. The findings described in this work provide valuable information regarding our understanding of virus neutralization and immune escape, which may help in the development of novel antiviral compounds.

**Supplementary Information:**

The online version contains supplementary material available at 10.1186/s13567-025-01505-z.

## Introduction

Rabbit hemorrhagic disease is a fatal and highly infectious disease of the European rabbit (*Oryctolagus cuniculus*) reviewed in [1]. The causative agent, rabbit hemorrhagic disease virus (RHDV) (species *Lagovirus europaeus*), belongs to the *Lagovirus* genus of the *Caliciviridae* family. Phylogenetic analysis has led to the classification of RHDV as a genogroup GI, which can be further divided into two genotypes, GI.1 and GI.2 [2]. GI.1 contains the variants GI.1 a–d, including the antigenic variant RHDV G1.1a (previously RHDVa), which is highly pathogenic and has different hemagglutination properties and monoclonal antibody binding patterns to GI.1b [3]. Since it emerged in 2010 [4, 5], GI.2 has spread worldwide and has replaced the older RHDV GI.1 strains in Australia and the Iberian Peninsula [6–9]. While the effects of RHDV GI.1 on Australian and European wild rabbit populations are clearly evident, the effects of GI.2 are more varied, with a substantial negative effect on Australian rabbits [10] and a mixed effect dependent on regional variation in Spain [11].

Unlike GI.1, RHDV GI.2 causes disease and death in kittens under 40 days of age [7]. Differences between the GI.1 and GI.2 isolates require modifications to diagnostic methods, as GI.2 shows modified hemagglutination properties and antigenic differences [5, 12]. Early GI.2 isolates demonstrated mortality rates of 20% and 50% in adults and young rabbits, respectively [5], whereas protection following exposure to GI.1 was only partial [13].

The RHDV capsid protein VP60 (also termed VP1) is the major viral antigen, and when produced in heterologous systems, it serves as an excellent recombinant vaccine candidate [14–18]. Structurally, VP60 is composed of three domains: the N-terminal arm (NTA), the shell (S) domain, and the protruding (P) domain. The P domain can be further divided into P1 and P2 subdomains, with P2 located at the outer exposed region of the virus capsid. The inner S domain and P1 subdomain are structurally conserved among several caliciviruses [19]. However, the predicted P2 structure is more variable, containing 7 loops (L1–L7) of various lengths that differ significantly among calicivirus P domains and may play a defining role in host tropism for each virus species [19, 20]. The average amino acid similarity between GI.1 and GI.2 VP60 is reported at 89.2% [21], which is reduced to 65.3% if only the 7 variable loop structures (L1–L7) present on the surface of the P2 subdomain are considered [12, 19, 21].

The calicivirus P2 subdomain is under selective pressure from the host immune response [22] and is also involved in binding host molecules on the cell surface. RHDV is thought to gain entry into the host via the upper respiratory or digestive tracts by binding to the carbohydrate moieties of histo-blood group antigens (HBGAs) displayed on epithelial cells [23, 24]. Polymorphisms of these molecules between species may explain host susceptibility but not virulence [25]. RHDV agglutinates human red blood cells (RBCs), and this interaction is often used for diagnosis and virus quantification. X-ray crystallography revealed that GI.2 P domain dimers contain an HBGA binding pocket at the dimeric interface [26]. This binding pocket includes amino acids from loops 3 and 7 in the P2 subdomain, with residues from both monomers contributing to HBGA binding.

MAbs with the ability to inhibit RBC agglutination by recognizing both internally buried and externally exposed epitopes of VP60 have been described [3, 27]. These antibodies constitute important tools for virus typing. Fine mapping of mAb epitopes has led to the identification of VP60 antigenic sequences [3, 27–29], and mAbs that show potential serotype (GI.1 and GI.2)-specific binding have also been identified [12, 20, 30, 31].

Two neutralizing mAbs against RHDV GI.1 have been described [3, 29] that recognize externally exposed conformational epitopes [3]. Structural analysis of the mAb-VLP complexes suggested possible mechanism(s) to explain the neutralizing capacity of the mAb E3 [29].

Mab 2D9 is GI.2 specific [30, 31], and previous studies have indicated the structural basis for 2D9 GI.2 specificity [32]. Here, we describe the further characterization of the interaction of the mAb 2D9 with the GI.2 capsid and P domain and demonstrate its neutralizing activity. MAb 2D9 escape mutant sequences from field samples indicate that mutations not directly implicated in antibody‒virus interactions can substantially affect binding capacity and should be monitored in future epidemiological studies.

## Materials and methods

### Virus stocks and sequences

Viral stocks or genome sequences from isolates belonging to GI.1 (GI.1b/RHDV isolate RHDV-Ast89, GenBank accession number Z29471), G1.1a (previously RHDVa GenBank accession number KF270630) and RHDV GI.2/b/(RHDVb/2 N-11, accession number KM878681 and RHDVb/2 Gal08/13 accession number ON854865) were used in this study.

### VLP and overlapping VP60 partial fragment production

The major structural protein (VP60) of RHDVGI.2/b variant N-11, RHDVGI.1a variant Gal09/12 or RHDVGI.1b/RHDV-Ast89 was expressed in *Spodoptera frugiperda* (Sf9) cells using a recombinant baculovirus expression system. To obtain recombinant baculovirus, Sf9 cell cultures were transfected with mixtures of BAC_10_:KO1629 linearized with the restriction enzyme *Bsu*36I (or *Eco*81I) and the transfer vector pTriEx containing the desired VP60 sequence, following a standard transfection protocol. The presence of VP60 in the cell extracts was analysed by western blot using polyclonal antibody sera against RHDV. Recombinant baculovirus stocks were cultivated in Sf9 cells grown in suspension and titrated on monolayers of the same type of cells. VLPs were purified by ultracentrifugation through a cesium chloride gradient [33] and the purified stocks were quantified using the Bradford method and densitometry following SDS-PAGE analysis.

Partial overlapping fragments of the GI.2 variant N11 VP60 were amplified from the construction vector pTriEx-VP60-Nav10/11 using the primers described in Table 1, and the amplicons were subsequently cloned and inserted into the vector pGex-2T using *Bam*HI or *Bsa*I and *Eco*RI restriction sites. The final plasmids were verified by restriction enzyme digestion and DNA sequencing (ABI PRISM 3130xl Genetic Analyser) using pGex 5’ and 3’ sequencing primers and then transformed into *E. coli* BL21 cells to produce GST-tagged VP60 fragments.

**Table 1:** **Sequences of the primers designed for VP60 fragments cloning**

Fragment	Name	Primer Sequence (5ʹ-3ʹ)ⁱ	Position in VP60
NTA	Ndom/*Bam*HI-5ʹ	GCATGCATGCGGATCCATGGAGGGCAAAGCCCGCGCGG	1–262
	Ndom/*Eco*RI-3ʹ	ACAGTGTACAGAATTCTGCCCGGGGCGTCTGCAACTGA	
S	Sdom/*Bsa*I-5ʹ	GTCGATTGCAGGTCTCGGATCCGGCGGTCCACCCCAACAAGTGG	166–822
	Sdom/*Eco*RI-3ʹ	CGTTCCAGTGGAATTCGCACGTAGAAAACCCACCGGGG	
P2	P2dom/*Bam*HI-5ʹ	GCCATTTGGTGGATCCACGAGCGCGATC	601–1370
	P2dom/*Eco*RI-3ʹ	ATGATGGGTGAATTCTTGCCAATAGGAGCGGCAG	
P1	P1dom/*Bam*HI-5ʹ	GGGTTGTTTGGATCCGCATCGGGTGTCATATCCACCC	1264–1740
	P1dom/*Eco*RI-3ʹ	GCATGCATGCGAATTCTCAGACATAAGAAAAGCCATTG	

ⁱ Underlined letters indicate additional restriction enzyme sites: *Bam*HI, *Eco*RI or *Bsa*I.

A full P domain fragment fused to GST was obtained by subcloning from pMalc2X-PDomNav10/11 [26] into the pGeX4T-1 vector using the restriction enzymes *Bam*HI and *Not*I.

### Mutagenesis of P domains

Sequences corresponding to mutant RHDV GI.2 P domains flanked by the restriction sites *Pst*I and *Not*I were ordered from Integrated DNA Technologies (IDT) or generated by overlapping PCR via primers, which included the desired loop substitutions (Table 2), and primers flanking the P domain sequence from pMalc2X-PdomNav10/11, pMalc2X-F (5ʹ-TCAGACTGTCGATGAAGC-3ʹ) and pMalc2X-R (5ʹGATGTGCTGCAAGGCGAT-3ʹ). The presence of restriction sites in pMalc2X-PdomNav10/11 [34] allowed the substitution of Nav10/11-Pdom for each mutant P domain in pMalc2X-derived vectors. Plasmid sequences were analysed by Sanger sequencing to ensure that only the desired changes were present.

**Table 2:** **Primers used for mutagenesis of P domain loops**

Name	Sequence (5ʹ-3ʹ)*	Length (nt)	Sense
L1-Ast89-F	CATGACCGTGGTAgTGCAAGCTATCCGGGTAaCAa CgcCAcTAATGTTCTGcAAtTtTGGTATGCCAGCGC	71	+
L1-Ast89-R	GCGCTGGCATACCAaAaTTgCAGAACATTAgTGgcG tTGtTACCCGGATAGCTTGCAcTACCACGGTCATG	71	-
L2-Ast89-F1	CTGTGGTATGCCAaCGCAGGTAGCGCAatCGA TAATCCGATTAGCCAGgTTGCACCGGATG	61	+
L2-Ast89-R1	CATCCGGTGCAAcCTGGCTAATCGGATTA TCGatTGCGCTACCTGCgTTGGCATACCACAG	61	**-**
L2-Ast89-F2	GAGCTTTGTTCCGTTTAaCGGCcCCggCaTTCCGgCC GCAGGTTGGGTTGGTTTTGG	57	+
L2-Ast89-R2	CCAAAACCAACCCAACCTGCGGcCGGAAtGccGGg GCCGtTAAACGGAACAAAGCTC	57	−
L3-Ast89-F	GGTATTTGGAATAGCAaCAgTGGTGCACCGaaTGT TACCACCGTGCAGGCATATGAACTGGGTTTTG	67	+
L3-Ast89-R	CAAAACCCAGTTCATATGCCTGCACGGTGGTAACA ttCGGTGCACCAcTGtTGCTATTCCAAATACC	67	−
L4-Ast89-F	CATATGAACTGGGTTTTGCAACAGGTGCACCGgGC AATCtGCAGCCGACCACCACCACCTCAGGTGC	67	+
L4-Ast89-R	GCACCTGAGGTGGTGGTGGTCGGCTGCaGATTGCc CGGTGCACCTGTTGCAAAACCCAGTTCATATG	67	−
L5-Ast89-F	GCCAAAAGCATTTATGcTGTTGtCACCGGTAcTgc TCAGaatcCCGCAGGTCTGTTTGTTATGGC	65	+
L5-Ast89-R	GCCATAACAAACAGACCTGCGGgattCTGAgcAgT ACCGGTGaCAACAgCATAAATGCTTTTGGC	65	−

^*^ Lowercase indicated the modified nucleotides respect to RHDVGI.2/b wild type sequence.

### Protein purification

Fusion proteins consisting of a His-tagged P domain-MBP bearing each of the loop mutants were produced in *E. coli* and purified following a published protocol [34]. The recombinant proteins were analysed by SDS‒PAGE, western blotting and dot blotting using standard protocols [17, 18].

### ELISA analysis

ELISAs using P domains as antigens were carried out by coating 96-well flat-bottom plates (Corning, Kennebunk, USA) with different amounts of protein per well (12.5, 25, 50 and 100 ng) in PBS, pH 7.5, overnight at 4 °C. The unbound antigen was discarded, and the plates were washed once with 200 µL/well of PBS containing 0.05% Tween 20 (PBS-T) and blocked for 1 h at room temperature with 200 µL/well of blocking solution (1% yeast extract in PBS-T). The plates were then washed five times with 200 µL/well of PBS-T. One hundred (100) microliters/well of 2D9 antibody diluted 8000 times in blocking solution was added and incubated at 37 °C for 1 h. The plate was washed 5 times, and then 100 µL of rabbit anti-mouse IgG Fab specific peroxidase-conjugated (SIGMA, Darmstadt, Germany) diluted 5000 times in blocking solution was added and incubated for 1 h at 37 °C. Finally, the plate was washed 5 times and incubated with 100 µL/well of the TMB liquid substrate system for ELISA (SIGMA-Aldrich) for 15 min in the dark. The reaction was stopped by adding 100 µL of 3 N sulfuric acid to each well. The optical density (OD) was measured at 450 nm on a Varioskan® Flash (Thermo Scientific).

### Surface plasmon resonance (SPR) measurements

SPR measurements were carried out with a SPRIT Autolab SPR instrument (Ecochemie, The Netherlands). The gold chips were cleaned with piranha solution (3 H_2_SO_4_ (95%):1 H_2_O_2_ (33%)) and then modified with a 1:3 mixture of 11-mercaptoundecanoic acid and mercaptohexanol overnight at 4 °C. The carboxylic groups on the chip were subsequently activated by three successive injections of a 1:1 mixture of 200 mM N-(3-dimethylaminopropyl)-N′-ethylcarbodiimide hydrochloride (EDC) and 50 nM N-hydroxysuccinimide (NHS) for 10 min. A 2 µg/mL solution of 2D9 in 10 mM NaAc (pH 5.5) was subsequently injected for different durations, ranging from 15–20 min, with the aim of obtaining different antibody binding densities. The reference channel was modified with BSA. After washing with the same buffer, the remaining activated carboxylic groups were blocked with a 1 M ethanolamine solution in PBS for 15 min. For affinity interaction monitoring, the association step was carried out by adding VLPs at appropriate concentrations in PBS with 0.005% Tween-20. After cleaning, a dissociation step of 10 min was used to obtain a stable signal. The difference between the dissociation and baseline values was related to the binding of the antibody to the VLP.

### Statistical analysis

The optical densities obtained in the mAb 2D9 reactions against the mutant P domains were normalized and corrected according to [35], and the percentages of the relative OD values with respect to the positive control (GI.2 P domain) were obtained. After the normality and variance homogeneity of the ELISA data were evaluated, the Tukey test or Student’s *t* test was used to investigate the significance of the differences between the studied variables. The data that did not show normality were analysed by the Kruskal‒Wallis test.

### Virus neutralization and in vivo challenge

New Zealand white rabbits were supplied by San Bernardo Farm (Navarra, Spain). The 30-day-old rabbits were housed individually in a biosecurity level 2 laboratory and kept under observation until the start of the treatment. The experimental procedures were approved by the Ethical Committee of the Principality of Asturias and authorized by the Regional Consejería de Agroganadería y Recursos Autoctonos del Principado de Asturias, Spain (authorization code PROAE 19/2014). The experiments were conducted following Directive 2012/63/EU.

Virus neutralization was carried out by mixing equal quantities (17.2 µg of purified IgG) of monoclonal antibodies 2D9 or 3A10 with diluted GI.2 isolate RHDV-Gal08/13 virus homogenate, and the mixtures were incubated for 45 min at 37 °C. Virus samples were separately treated with 2D9 or 3A10 antibodies and injected into two RHDV antibody-negative rabbits to assess their putative neutralizing effects. Two animals were inoculated with the 2D9-treated virus, and two were inoculated with the virus-3A10 mixture. After challenge, the clinical state of the animals was assessed twice per day. After euthanasia or death, the rabbits were physically examined and necropsied. The necropsy analysis included examination of internal organs for gross lesions or symptoms of RHDV infection (specifically, the lungs, trachea, liver, spleen, and intestine) [36].

## Results

### MAb 2D9 reactivity to VLPs and overlapping VP60 fragments in dot and western blots

**Figure 1:**
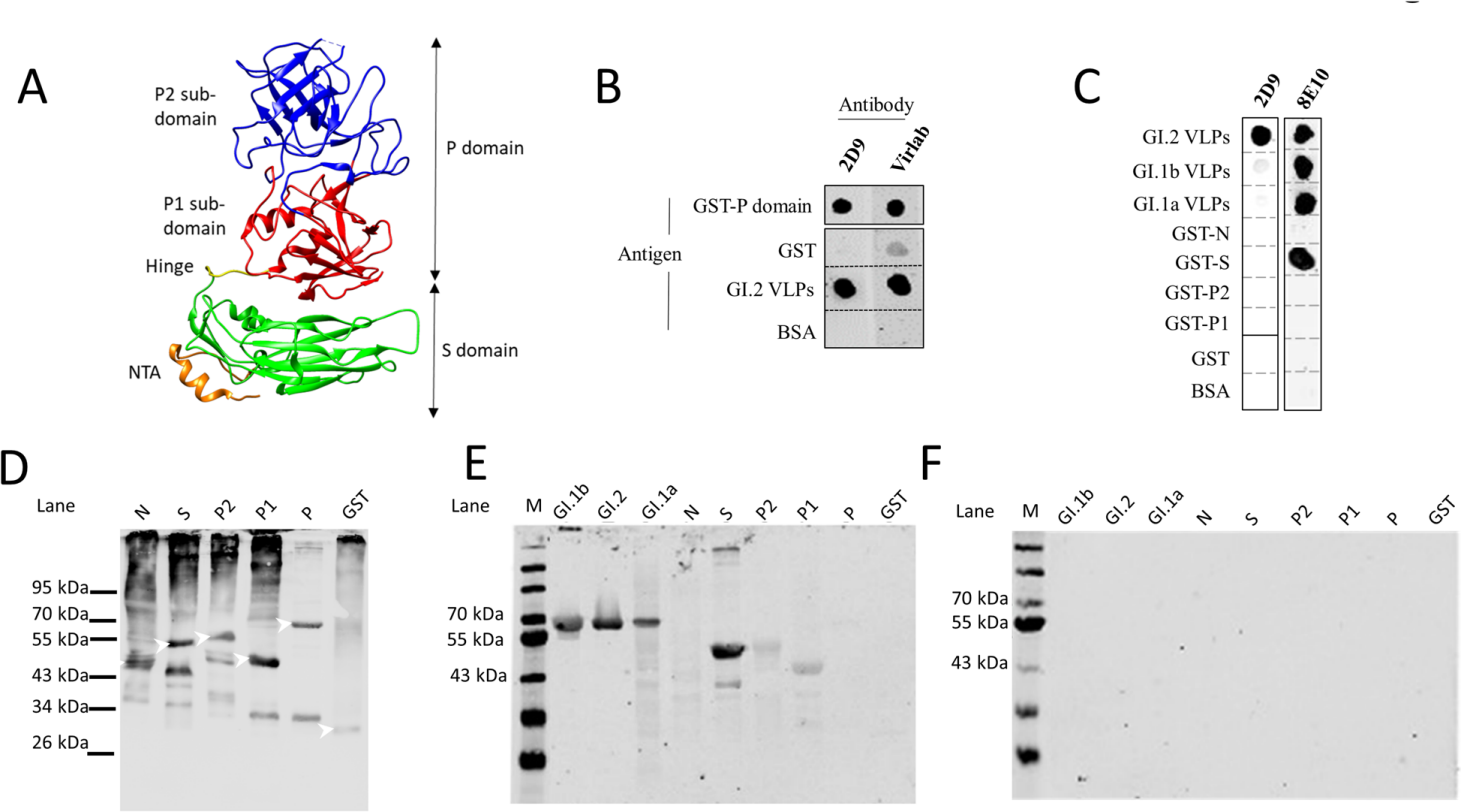
**Prediction model of VP60 organization and analysis of 2D9 binding specificity.** (A) Structural model showing the domain organization of the VP60 isolate GI.2 (adapted from Wang et al., 2013) based on the RHDVGI.2 structure pdb number 9JJJ [50]. The 3 major domains (N (N-terminal arm; in orange), S (shell; in green) and P (protruding)) and the hinge region (yellow) are indicated. The P domain is further divided into P1 and P2 subdomains (red and blue, respectively). (B) Dot blot analysis of the GST-P domain, GST, and GI.2 VLPs and BSA were generated via the mAb 2D9 and anti-RHDV polyclonal sera denominated Virlab. (C) Dot blot analysis using mAbs 2D9 and 8E10 to detect VLPs (GI.2, GI.1b and GI.1a) and GST-VP60 fusion fragments corresponding to the N, S, P2, and P1 domains as antigens. Negative controls containing GST and BSA are also shown. (D) Western blot analysis of GST-GI.2 fusion proteins developed with an anti-GST antibody. Lane N: GST-N (predicted molecular mass of 35.9 kDa); Lane S: GST-S (50.6 kDa); Lane P2: GST-P2 (52.5 kDa); Lane P1: GST-P1 (42.5 kDa); Lane P: GST-P (61 kDa). The white arrowheads indicate protein bands corresponding to the fusion proteins. (E) and (F) Western blot analyses using mAbs 2D9 (E) and 8E10 (F) for the detection of RHDV VLPs (G1.2; G1.1a; G1.1b) and partial overlapping fragments (N, S, P, P1, and P2) of GI.2 VP60 is produced as a GST-fusion protein.

To investigate mAb 2D9 specificity, analyses were carried out using VLPs and a series of VP60 fragments in dot blot assays. A model representing the structural organization of RHDV VP60 on the basis of the GI.2 structure (9JJJ) indicating the 3 major domains is shown in Figure 1A. In addition to the analysis of VLPs, five GST-fusion proteins containing individual GIs were identified.2 VP60 domains or subdomains, N (amino acid residues 1–87), S (56–274), P (230–569), P1 (422–579), and P2 (204–457), were evaluated for 2D9 binding. As expected, mAb 2D9 specifically bound to RHDV GI.2 VLPs (Figures 1B and C) and did not bind to GI.1a or GI.1b VLPs (Figure 1C). Furthermore, 2D9 bound full-length GST-GI.2 the P domain in the dot plot (Figure 1B) did not react to GST-GI.2 P1 or P2 subdomains or fragments of GST-GI.2 N or S domains (Figure 1C). An additional mAb, termed 8E10, was shown to bind to VLPs of all three RHDV types (GI.2, GI.1a or GI.1b) by dot blotting and to the GST-GI.2-S fusion protein (Figure 1C). Therefore, dot blot analyses confirmed that 2D9 is GI.2 specific and recognizes an epitope present on full-length P domains.

Western blotting with a GST-specific mAb was used to confirm the molecular masses of the GST fusion fragments (Figure 1D). Similar electrophoretic and western blot analyses demonstrated that 2D9 did not recognize a linear epitope as GI did.2 VLPs or GST-GI.2-VP60 fragments were recognized under these conditions (Figure 1E), suggesting that the mAb 2D9 binds a conformational or discontinuous epitope. A duplicate blot prepared with the mAb 8E10 (Figure 1F) confirmed that the mAb 8E10 reacted with all three denatured VLP types and the GST-GI.2-S fragment (Figure 1F), suggesting that it recognized a linear epitope within the VP60 shell (S) domain.

### Binding affinity of 2D9 for GI.2 VLPs

**Figure 2:**
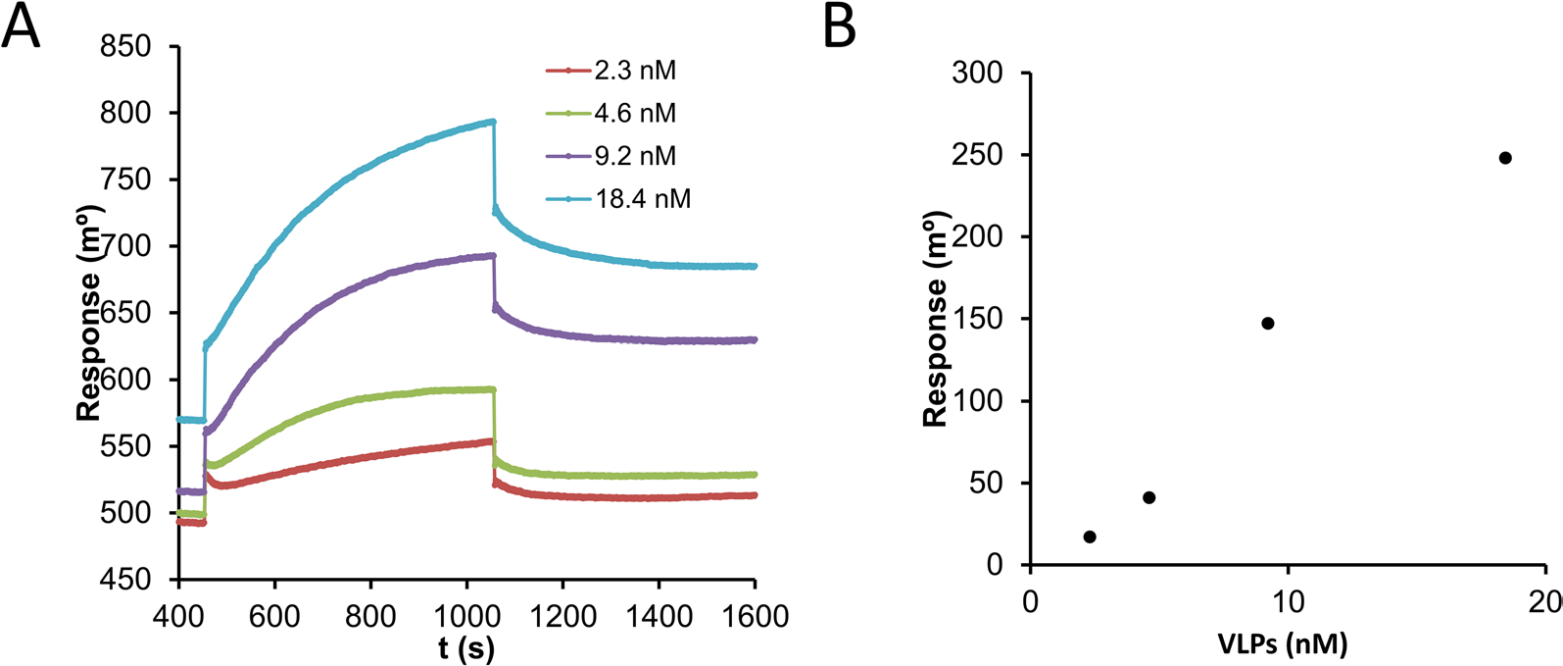
**SPR analysis of 2D9 binding to GI.2 VLPs.** **a** Overlay of the SPR sensograms obtained after successive incubations with increasing concentrations of VLPs, using an SPR disk modified with 1.918 ng/mm^2^ 2D9 IgGs; **b** Corresponding binding isotherm from which a K_d_ = 9 ± 2 nM is estimated (hill fitting).

SPR was used to investigate the binding affinity of mAb 2D9 to GI.2 VLPs. For this purpose, VLPs were injected over 2D9 IgGs immobilized on gold chips, and the resulting SPR sensograms, obtained after successive incubations with increasing concentrations of VLPs (Figure 2A), were used to construct binding isotherms (Figure 2B).

The SPR binding responses were analysed via the Langmuir absorption and Hill models (Table 3). K_d_ value of mAb 2D9 binding to GI.2 VLPs were found to be in the range of 9 ± 2 nM using Hill fitting (Figure 2).

**Table 3:** **VLP-2D9 binding affinity determination via the Langmuir absorption and Hill models**

[2D9] immobilized	Langmuir absorption model		Hill model		
	K/nM	r	K/nM	n	r
0.542 ng/mm^2^	7 ± 2	0.994	5 ± 2	1.2 ± 0.2	0.995
0.989 ng/mm^2^	11 ± 2	0.9981	6.5 ± 0.7	1.16 ± 0.05	0.9996
1.918 ng/mm^2^		No fit	9 ± 2	2.4 ± 0.5	0.9934

n: stoichiometry of binding, r: correlation coefficient/quality of fit, K: Kd in nM.

### Production of the RHDVGI.2 mutant P domains

Externally exposed P domain loops are involved in RHDV GI.1 neutralizing antibody binding [19] and 2D9 GI.2 P domain interactions [32]. To further investigate the residues required for 2D9 binding, considering that this mAb did not recognize GI.1b VLPs, we substituted GI.2 L1 to L5 loops individually, or in combination, for the equivalent GI.1b loops in the backbone of the GI.2 P domain. Table 4 shows a summary of the loop mutant constructs, indicating the position of mutations within the VP60 sequence and the number of substitutions contained in each. The number of amino acid residue substitutions in loop replacement constructs varied from 2–8 for individual loop exchanges and from 9–16 for the replacement of two or three loops simultaneously.

**Table 4:** **Summary of mutations identified in GI.2 P domain for 2D9 binding analysis**

Construct	Mutated loop sequence	Position in VP60	No of amino acid changes
L1´	SASYPGNNATNVLQF	301–315 (L1)	7
L2´	NAGSAIDNPISQVAPDGFPDMSFVPFNGPGIPAA	319–352 (L2)	8
L3´	WNSNSGAPNVTTVQA	361–375 (L3)	3
L4´	TGAPGNLQ	382–389 (L4)	2
L5´	IYAVVTGTAQNPA	404–416 (L5)	7
L1´L4´	SASYPGNNATNVLQF/TGAPGNLQ	301–315/382–389	9
L4´L5´	TGAPGNLQ/IYAVVTGTAQNPA	382–389/404–416	9
L1´L4´L5´	SASYPGNNATNVLQF/TGAPGNLQ IYAVVTGTAQNPA	301–315/382–389/404–416	16
N387D	TGAPSDPQ	387	1
N387A	TGAPSAPQ	387	1
AT414-15	IYGVATGINQTAA	414–415	2

Underlined residues indicate amino acid changes with respect to wild type GI.2.

Partial alignments including loop regions and secondary structure models of each modified P domain loop indicating residue changes with respect to the GI.2 The VP60 protein is shown in Additional file 1.

### Binding of 2D9 to P domain loop-swap mutants

**Figure 3:**
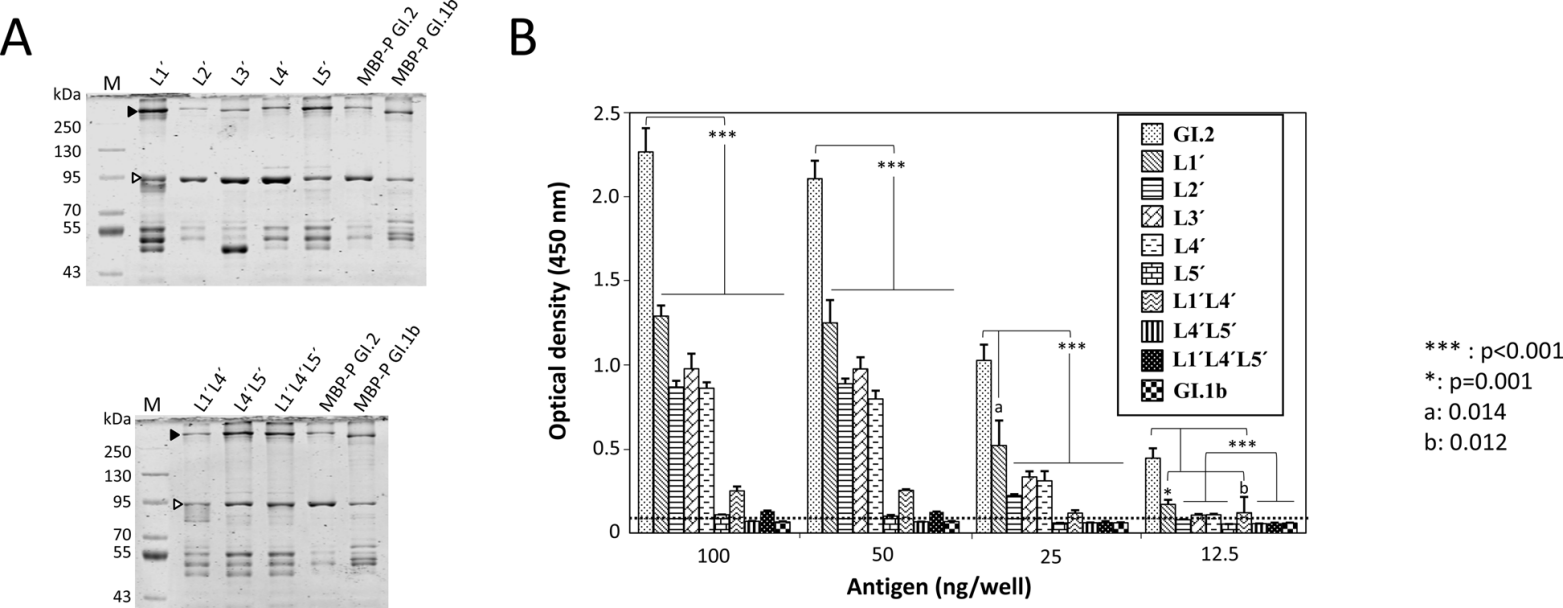
**Dimerization capacity and 2D9 binding of P domain loop swap mutants.** **A** Analysis of the dimerization capacity of P domain mutants using polyacrylamide gel electrophoresis under nondenaturing conditions and western blotting with an anti-His tag monoclonal antibody. Each lane name corresponds to the P domain mutant analysed. The full-length GI.2 and GI.1b P domains (as MBP fusions) are shown as controls. Arrowheads indicate bands corresponding to fusions of the P domain in monomeric (white) and homodimeric (black) forms. M: molecular mass markers. **B** ELISA of the interaction of 2D9 with the GI.2 wild-type P domain or with single or multiple loop substitutions (the name of each loop mutant is shown in the inset). The mean values and standard deviations of the optical densities are presented for each sample analysed in triplicate. The cut-off value is indicated by a horizontal discontinuous line. ***: *p* < 0.001; *: *p* = 0.001, a: 0.014 and b: 0.012.

Full-length folded P domains were required for the 2D9 interaction (Figures 1B and E). We hypothesized that dimerization may be important for binding; therefore, loop-swap mutant P domains were analysed for dimerization by nondenaturing polyacrylamide gel electrophoresis (PAGE) (Figure 3A). All the samples analysed (mutant or wild type) presented a protein band with a calculated molecular mass of 85 kDa, corresponding to monomeric forms (white arrowheads), and a protein band with lower electrophoretic mobility (black arrowheads), indicating dimeric forms of the mutant P domains (Figure 3A). Additionally, lower molecular mass bands corresponding to unspecified degradation products were also detected in all lanes.

Analyses of 2D9 binding to the wild-type or modified P domains were carried out via indirect ELISA, with the GI.2 and GI.1b P domains used as positive and negative controls, respectively (Figure 3B). The results indicate that all P domains bearing one or several loops substituted with the corresponding GI.1b P domain sequences had reduced interactions with mAb 2D9 (Figure 3B). This was particularly evident for the single loop 5 substitution (2% binding), the simultaneous substitution of loops L4’L5’ (0.06%) and loops L1’L4’L5’ (2.68%), where the detected signal was similar to the cut-off values determined by ELISA (Figure 3B).

Statistical analyses revealed that, in comparison with the wild-type GI.2 P domain 2D9 interaction was significantly reduced in L5-modified P domains, either individually or together with one (L4´L5´) or more additional loop (L1´L4´L5´), with respect to other single loop mutants (L1´, L2´, L3´ and L4´).

### Mutants based on natural 2D9 escape variants

**Figure 4:**
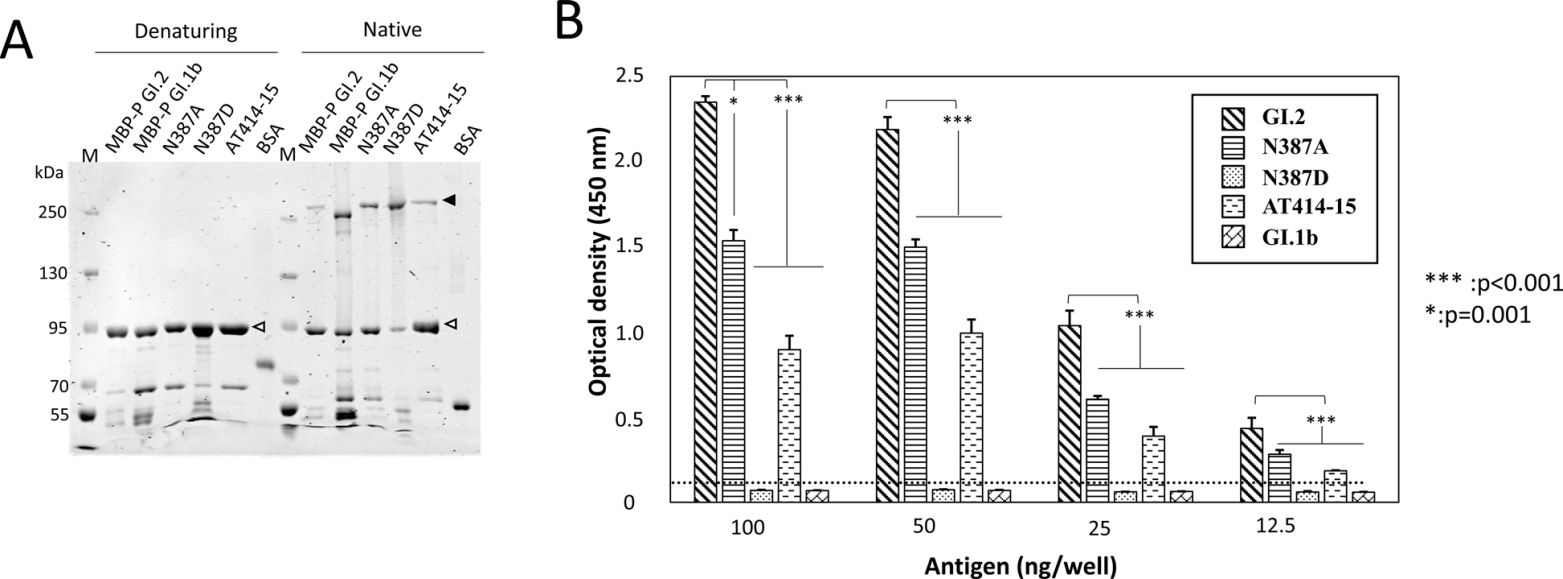
**Dimerization capacity and 2D9 binding of P domain point mutants.** **A** Analysis of the dimerization capacity of P domain mutants using polyacrylamide gel electrophoresis under denaturing or native conditions. Each lane name corresponds to the P domain mutant analysed. MBP-P GI.2 and GI.1b are shown as positive controls, and BSA is used as a negative control. Arrowheads indicate bands corresponding to fusions of the P domain in monomeric (white) and homodimeric (black) forms. M: molecular mass markers. **B** ELISA analysis of the interaction of 2D9 with the GI.2 wild-type or mutant P domains (each mutant shown in the inset). The mean values and standard deviations of the optical density are presented for each sample analysed in triplicate. The cut-off value is indicated by a horizontal discontinuous line. *** *p* < 0.001; * *p* = 0.001.

Three additional P domain mutants were designed on the basis of the identification of naturally occurring 2D9 escape mutants. Two natural RHDV GI.2 isolates, RHDV-Ger06/12–2 and RHDV-Ler11/16–1, from two independent outbreaks detected in Spain showed no reactivity to the monoclonal antibody 2D9, although these isolates were classified as GI.2 on the basis of VP60 sequence analysis (data not shown). Sequence analysis of the RHDV-Ger06/12–2 isolate revealed the substitution of an asparagine residue (N^387^) with an aspartic acid residue (D^387^) mapping to loop 4 of the P2 subdomain. This substitution was not present in an additional isolate from an animal affected in the same outbreak that was reactive with 2D9. The RHDV-Ler11/16–1 isolate contained mutations in both loops 4 and 5. The mutations caused predicted amino acid changes in loop 4 (S^386^/R^386^) and loop 5 (T^409^/N^409^) and (A^414^T^415^/T^414^A^415^). On the basis of these findings, 3 additional P domain mutants were included in this study: N387D, N387A and AT414-415NP. The ability of these engineered P domains to form dimers (Figure 4A) and their 2D9 binding (Figure 4B) was analysed by native PAGE and ELISA, respectively. All the studied P domain mutants formed dimers (Figure 4A), and the ELISA data indicated that the construct including the N387D substitution was not recognized by 2D9, while the signals obtained for the other mutants were significantly reduced to 64% (N387A) and 36% (AT414-15) (Figure 4B).

### Determination of 2D9 monoclonal neutralizing capacity

**Figure 5:**
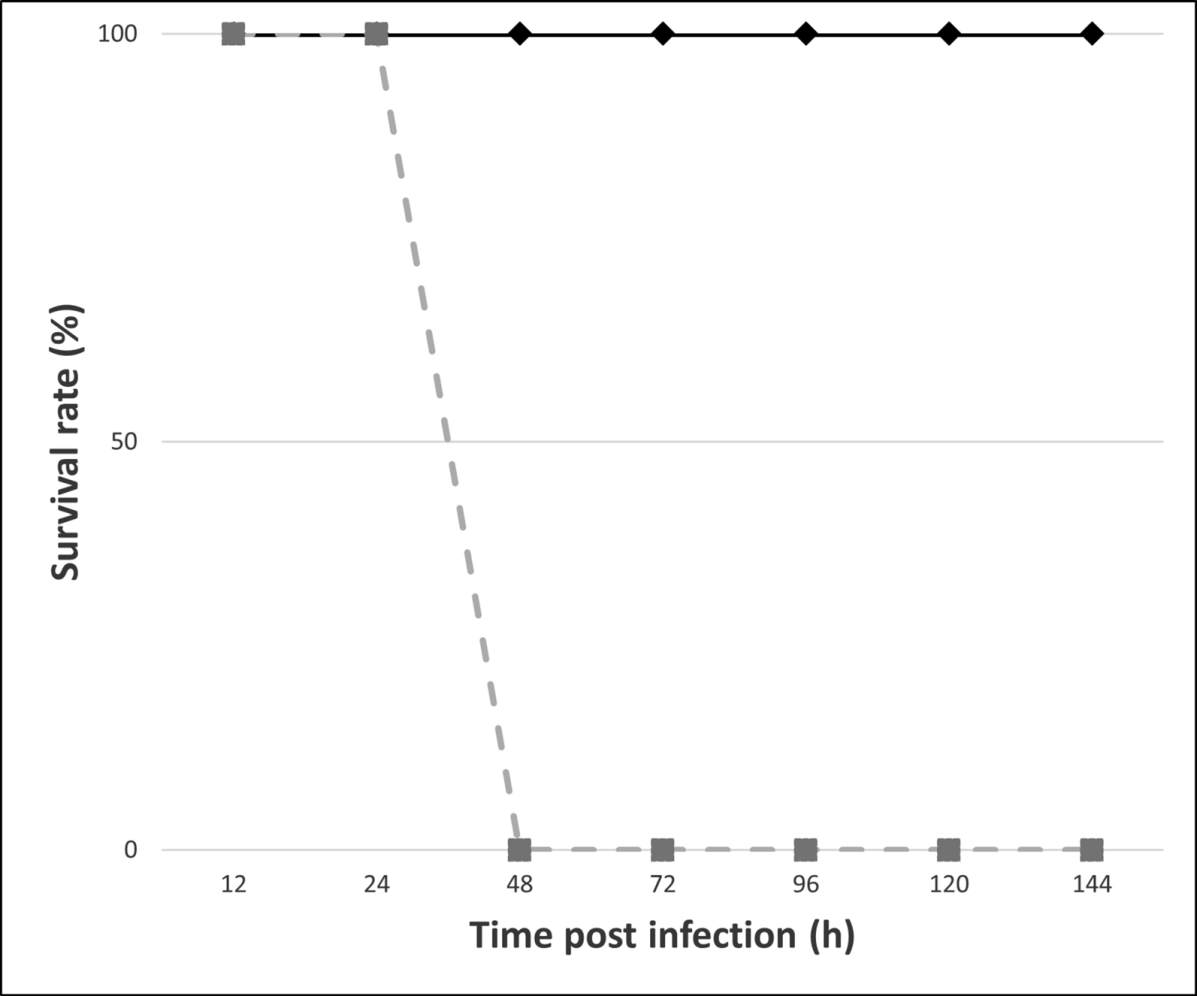
**In vivo neutralization study**. The graph shows the survival rate (%) analysis of experimentally infected rabbits with the GI.2 isolate RHDV-Gal08/13 previously incubated with 2D9 (black diamonds) or 3A10 (grey squares) mAbs.

Given the diagnostic potential of the mAb 2D9 and the fact that we detected natural escape mutants in infected rabbits, we were interested in determining whether 2D9 was capable of neutralizing virus infection. As this virus cannot be readily propagated in cell culture, an animal experimental challenge was carried out. A dose of 1000 × LD_50_ of RHDV GI.2 was incubated separately in vitro with monoclonal antibodies 2D9 (GI.2 specific) or 3A10 (recognizing both the GI.1 and GI.2 serotypes) [31]. The mixtures were subsequently used to infect two rabbits each (see Materials and methods). No clinical signs were observed in either of the treatment groups before 48 h post infection (hpi). At 48 h, the two rabbits challenged with the 3A10-treated virus died, showing signs of RHD. In the case of animals infected with 2D9-treated virus, both animals survived challenge and were healthy at 144 hpi (Figure 5).

## Discussion

MAbs against RHDV have proven essential in deciphering the structure of VP60 and in the development of diagnostic tools [27, 28, 37]. More recently, genotype-specific mAbs have been identified [12, 20, 31]. Additionally, a GI.1-specific conformational epitope and two linear epitopes have been described in the variable regions of the P1 subdomain and loop 1 of the P2 subdomain [12, 20].

The mAb 2D9 was used in the development of a GI.2-specific point-of-care diagnostic test [30]. Dot blot and western blot analyses (Figure 1) to map the 2D9 epitope indicated that this antibody recognizes a complex structure rather than a linear amino acid sequence, which is in agreement with the structural data [32]. SPR demonstrated that the binding affinity of 2D9 was strong, with a similar affinity determined via isothermal titration calorimetry (ITC) (K_d_ of 10.8 nm) [32]. Blot analyses revealed that only VLPs or full P-domains under native conditions (dot blot) were recognized, confirming that the 2D9 epitope was conformational or discontinuous. It seems reasonable to assume that the P1 and P2 subdomains might not fold properly when produced separately, as the P subdomain amino acid sequences are not contiguous. Bearing in mind the conformation requirements for 2D9 binding, the structurally flexible, externally exposed loops of the P2 subdomain, containing multiple differences between genotypes [26], were targeted for our mutagenic analyses.

Binding analysis of loop swap mutants indicated that all studied loops (L1 to L5) contributed to the P domain-2D9 interaction. However, the most drastic reduction was observed after the substitution of loop 5, which completely abrogated the 2D9 interaction. Loop 5 is 13 amino acid residues in length and contains 7 amino acid differences between the GI.2 and GI.1 sequences. Likewise, all loop mutant combinations that included L5 substitution drastically affected P domain binding to 2D9. While individual L1 or L4 substitutions resulted in reductions of 57% and 37%, respectively, the combination of L1 and L4 led to greater than 90% reduction in binding. These results suggest that in addition to L5, L1 and L4 also play relevant roles. This might be due to direct interactions of 2D9 with L1 and L4 or due to perturbations in proximal structures that affect binding. Residues at distant positions with respect to binding epitopes can produce conformational perturbations in viral capsids, which affect the binding site of neutralizing antibodies [38, 39]. Therefore, mutations at positions not directly involved in the 2D9 interaction could disturb the conformation of the GI.2 neutralizing epitope, thereby limiting the flexibility of the epitope or driving the conformation towards a weak binding structure [40].

By analysing samples from two separate RHDV outbreaks on Spanish rabbit farms, we detected GI.2 confirmed virus isolates that were not reactive to mAb 2D9. The presence of 2D9 escape mutants during farm outbreaks suggested selective pressure on mutations in the 2D9 epitope region. Interestingly, both of these GI.2 isolates presented mutations in loops L4 and L5 (isolate 1 N387D loop 4 of the P2 subdomain; isolate 2 S386R loop 4 and T409N, AT414-15TA loop 5 of the P2 subdomain). The analysis of the single amino acid mutant N387D indicated that this single change was sufficient to abolish 2D9 binding. However, the N387A mutant had a 64% reduction in binding. The GI.2 The P domain used in this study contains electronegative pockets on its surface [26], and N387D substitution changes the nature of the charge in this region, likely contributing to the complete loss of binding. These data indicate the relevance of this L4 residue position for 2D9 interaction with the P domain. The binding of the mutant AT414-15NP P domain was drastically reduced, indicating that these residues are also relevant for supporting the role of L5 in the 2D9 interaction. In a structural analysis of 2D9-P domain binding, these loop regions, particularly N387, were also shown to be involved in antibody engagement [32].

The identification of calicivirus antigenic determinants has been the subject of numerous studies [38, 41–46]. Externally exposed loop 1 of the P2 subdomain has been identified as a potential target for viral neutralization of RHDV GI.1 [19]. The relationships between hypervariable regions of P domain-exposed loops from caliciviruses and neutralizing epitopes have been previously demonstrated by neutralizing-antibody escape mutant analyses [47, 48]. However, the study of the antigenic characteristics of RHDV has been limited by the lack of a reliable cell culture system for this virus. Consequently, studies carried out to characterize potential neutralizing antibodies require in vivo challenge experiments. Our preliminary challenge study demonstrated the ability of the 2D9 antibody to prevent infection in experimentally challenged rabbits. The relevance of the epitope recognized by 2D9 in virus neutralization is further supported by the identification of escape mutants not recognized by this mAb. Taken together, these results support that the P domain of RHDV GI.2 VP60 and, more precisely, P2 subdomain loops 1, 4 and 5 contain the key residues involved in the conformation of the neutralizing epitope recognized by mAb 2D9.

Previous studies have shown that amino acid residues are located in the external variable RHDV GI.2 P domain loops 3 and 7 are directly involved in binding to HBGAs [26], allowing virus interaction with host cells. Considering that our data indicate that 2D9 mAb binding requires a very complex structure involving at least loops L1 to L5, binding of the mAb 2D9 might directly or indirectly interfere with key cell–virus recognition events, such as HBGA binding, indicating a putative mechanism of action for this mAb, as has been suggested for murine norovirus [43]. The recent description of a cell culture model for RHDV using liver organoids [49] may provide a suitable experimental system to further test these conjectures.

## Supplementary Information

**Additional file 1. Partial amino acid sequence alignments and P domain structure models showing GI.2, GI.1b and selected loop amino acid substitutions.** L1 (red), L2 (yellow), L3 (magenta), L4 (orange) and L5 (blue).

## Data Availability

All data and material are available upon reasonable request.
