# Supplementary figures and images for: Epitope mapping of a neutralizing antibody against rabbit hemorrhagic disease virus GI.2
Source: Vet Res. 2025 Apr 2;56:74. doi: 10.1186/s13567-025-01505-z (PMC11963670; doi:10.1186/s13567-025-01505-z)

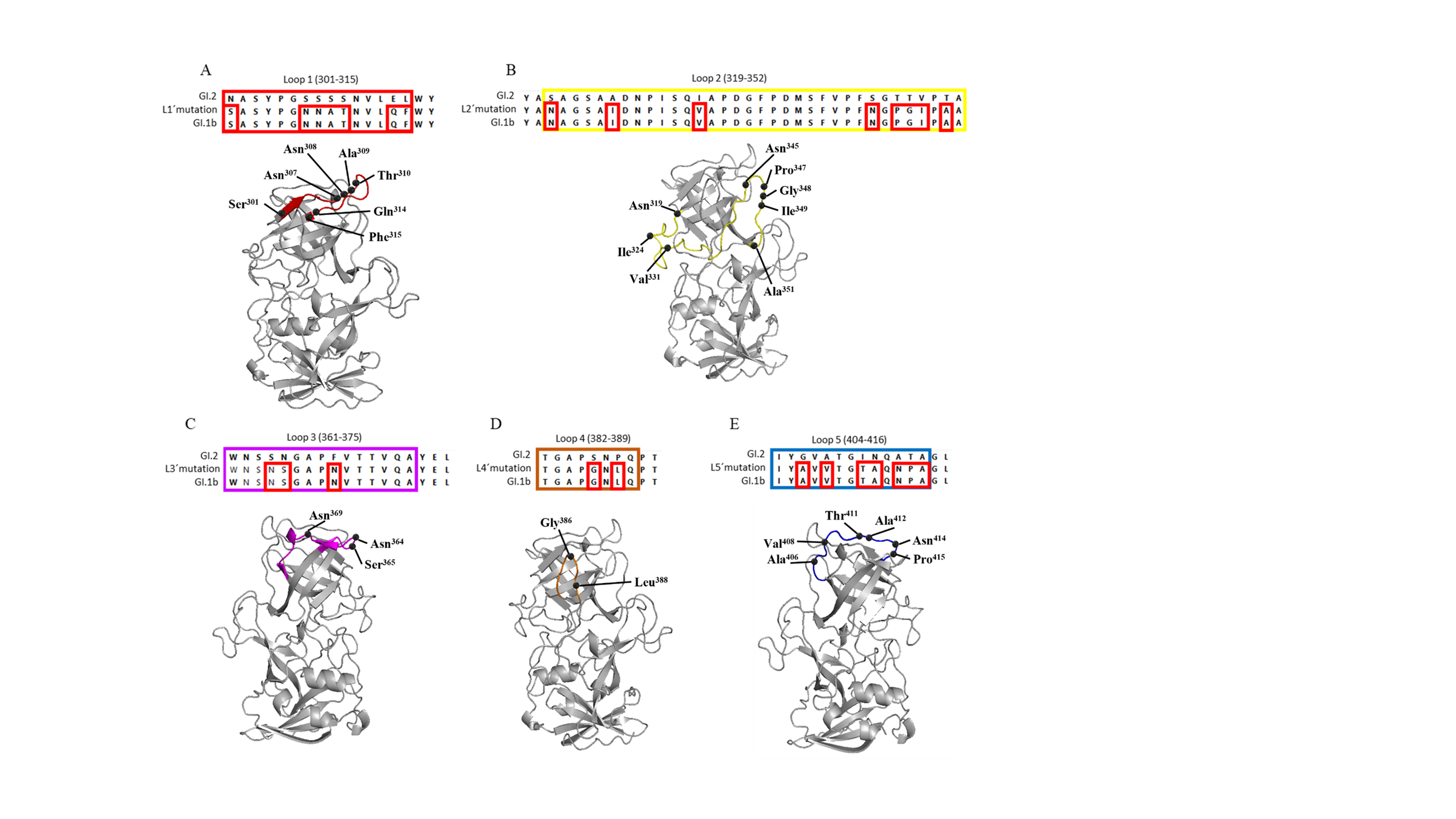

Supplement: Supplementary file 1 — Additional file 1. Partial amino acid sequence alignments and P domain structure models showing GI.2, GI.1b and selected loop amino acid substitutions. L1 (red), L2 (yellow), L3 (magenta), L4 (orange) and L5 (blue). [file 13567_2025_1505_MOESM1_ESM.tif]
